# Acupuncture Therapy for Functional Effects and Quality of Life in COPD Patients: A Systematic Review and Meta-Analysis

**DOI:** 10.1155/2018/3026726

**Published:** 2018-05-20

**Authors:** Jiajia Wang, Jiansheng Li, Xueqing Yu, Yang Xie

**Affiliations:** ^1^Dongzhimen Hospital, Beijing University of Chinese Medicine, Beijing 100700, China; ^2^Collaborative Innovation Center for Respiratory Disease Diagnosis and Treatment & Chinese Medicine Development of Henan Province, Henan University of Chinese Medicine, Zhengzhou, Henan 450046, China; ^3^Henan Key Laboratory of Chinese Medicine for Respiratory Disease, Henan University of Chinese Medicine, Zhengzhou, Henan 450046, China; ^4^Department of Respiratory Diseases, The First Affiliated Hospital of Henan University of Chinese Medicine, Zhengzhou, Henan 450000, China

## Abstract

**Objective:**

This study aimed to evaluate the efficacy and safety of acupuncture therapy (AT) for improving functional effects and quality of life in COPD patients.

**Methods:**

PubMed, Embase, Cochrane Library, Web of Science, Chinese Biomedical Literature Database (CBM), China National Knowledge Infrastructure (CNKI), Chongqing VIP (CQVIP), and Wanfang Data were searched. The randomized controlled trials (RCTs) evaluating the effect of AT on COPD patients were included. Primary outcome measures included six-minute walk distance (6MWD) and St. George's Respiratory Questionnaire (SGRQ). Study selection, data extraction, and risk of bias assessment were independently conducted, respectively. Statistical analysis was conducted by RevMan software (version 5.3) and Stata software (version 12.0).

**Results:**

Nineteen studies (1298 participants) were included. 6MWD improved more (MD: 47.84; 95% CI: 23.33 to 72.35; *Z* = 3.83, *P* = 0.0001) and effective rate was higher (OR: 2.26; 95% CI: 1.43 to 3.58; *Z* = 3.48, *P* = 0.0005) in the experimental group compared to the control group. Symptom domain scores (MD: −24.86; 95% CI: −32.17 to −17.55; *Z* = 6.66, *P* < 0.00001), activity domain scores (MD: −16.52; 95% CI: −22.57 to −10.47; *Z* = 5.36, *P* < 0.00001) and impact domain scores (MD: −13.07; 95% CI: −17.23 to −8.92; *Z* = 6.16, *P* < 0.00001) of SGRQ in the experimental group improved more compared to the control group. There was no significant improvement in SGRQ total scores between two groups. The improvement of FEV_1_ was not significant between two groups, yet subgroup analysis showed that patients treated with AT adjunctive to other treatments improved more in FEV_1_ (MD: 0.41; 95% CI: 0.28 to 0.54; *Z* = 6.01, *P* < 0.00001) compared to those treated with other treatments alone.

**Conclusion:**

AT may be effective in improving functional effects and quality of life in COPD patients. Besides, AT may also improve pulmonary function of patients with COPD. However, further high-quality RCTs are needed to confirm the efficacy and safety of AT for COPD patients.

## 1. Introduction

Chronic obstructive pulmonary disease (COPD), a leading cause of morbidity and mortality, is characterized by progressive airflow obstruction, airway inflammation, and systemic effects or comorbidities and is projected to be the third leading cause of death worldwide by 2030 [1, 2]. Since breathlessness, exercise limitation, and health status impairment broadly exist in patients with COPD, effective measures should be taken to improve symptoms, exercise tolerance, and health status based on an individualized assessment of disease [1]. Although appropriate pharmacologic therapy has effect in reducing COPD symptoms and the frequency and severity of exacerbations and improving health status and exercise tolerance [1], its cost and adverse effects can never be ignored.

Acupuncture therapy (AT), one of the most popular treatments in alternative medicine, has been proven to be cost-effective and safe in many conditions [3–5]. However, there is limited evidence concerning its efficacy and safety. One previous review showed that AT might result in clinically important improvements in quality of life and dyspnea of COPD patients, but it is outdated [6]. Moreover, the interventions of included studies involved point application therapy, acupressure, and transcutaneous electrical stimulation over acupuncture points (Acu-TENS), and these techniques may not genuinely reflect the efficacy of AT based on theories of traditional Chinese medicine. Therefore, the current review aims to evaluate the efficacy and safety of AT for improving functional effects and quality of life in COPD patients.

## 2. Methods

### 2.1. Inclusion and Exclusion Criteria

We included randomized controlled trials (RCTs) in which the effects of AT on COPD patients were evaluated.

Participants had COPD defined as a clinical diagnosis of COPD, with a postbronchodilator fixed ratio of forced expiratory volume in 1 second (FEV_1_)/forced vital capacity (FVC) < 0.70 measured by spirometry, and those who had an acute exacerbation within four weeks before the study were excluded.

The intervention included AT, such as manual acupuncture, electroacupuncture, auricular acupuncture, and warm acupuncture, yet noninvasive techniques, such as single moxibustion, acupressure, point application, laser acupuncture, or Acu-TENS, were excluded.

Primary outcome measures included any of the following: (i) six-minute walk test/distance (6MWT/6MWD) [26] and (ii) St. George's Respiratory Questionnaire (SGRQ) [27]. Secondary outcome measures included any of the following: (i) FEV_1_, (ii) modified Medical Research Council dyspnea scale (mMRC) [28], (iii) effective rate, and (iv) adverse effects.

### 2.2. Literature Search

PubMed, Embase, Cochrane Library, Web of Science, Chinese Biomedical Literature Database (CBM), China National Knowledge Infrastructure (CNKI), Chongqing VIP (CQVIP), and Wanfang Data were searched from their inception to 31 December 2017. We developed detailed search strategies for each electronic database without language restrictions. Reference lists of eligible studies and previous systematic reviews were also reviewed to identify further eligible studies.

### 2.3. Study Selection

Two review authors (Yang Xie and Xueqing Yu) independently examined titles and abstracts retrieved from the search and selected all potentially eligible studies. Then these full-text articles were obtained and the same review authors reviewed them independently against the inclusion and exclusion criteria. A third review author (Jiansheng Li) acted as an arbiter when consensus could not be reached.

### 2.4. Data Extraction

Data extraction was independently conducted by two review authors (Yang Xie and Xueqing Yu) using a standardized data extraction sheet, involving information of authors, year of publication, study design, participants, intervention, comparator, and outcomes, with a third review author (Jiansheng Li) acting as an arbiter when disagreements existed between Yang Xie and Xueqing Yu.

### 2.5. Assessment of Risk of Bias

Methodological quality was evaluated using the Cochrane tool for assessing risk of bias in RCTs [29]. Two review authors (Yang Xie and Xueqing Yu) independently assessed and scored each study with a third review author (Jiansheng Li) acting as an arbiter when disagreements existed.

### 2.6. Statistical Analysis

Statistical analysis was conducted by RevMan software (version 5.3) [30] and Stata software (version 12.0; StataCorp LP, USA). We summarized data using odds ratio (OR) with 95% confidence intervals (CI) for dichotomous outcomes and mean difference (MD) with 95% CI for continuous outcomes. If the data could not be combined into a meta-analysis, we summarized them in the text. We used a *χ* ^2^ test to estimate heterogeneity of both the MD and OR. Further analysis was performed using the* I* ^2^ test. A random-effect model was used to interpret the results if heterogeneity was statistically significant, whereas a fixed-effect model was used if heterogeneity was not statistically significant. We regarded heterogeneity as substantial when* I* ^2^ was greater than 50% or a low *P* value (*P* < 0.10) was reported for the *χ* ^2^ test [31]. When more than 10 studies were included in the meta-analysis, we would investigate publication bias by funnel plots. In addition, a metaregression analysis was performed to explore potential associations between effect size and covariates of interest (publication year, region, intervention forms, sample size, and treatment period). If necessary, we conducted subgroup analysis to assess whether the treatment effects were different in different subgroups.

## 3. Results

### 3.1. Literature Search and Study Selection

**Figure 1:**
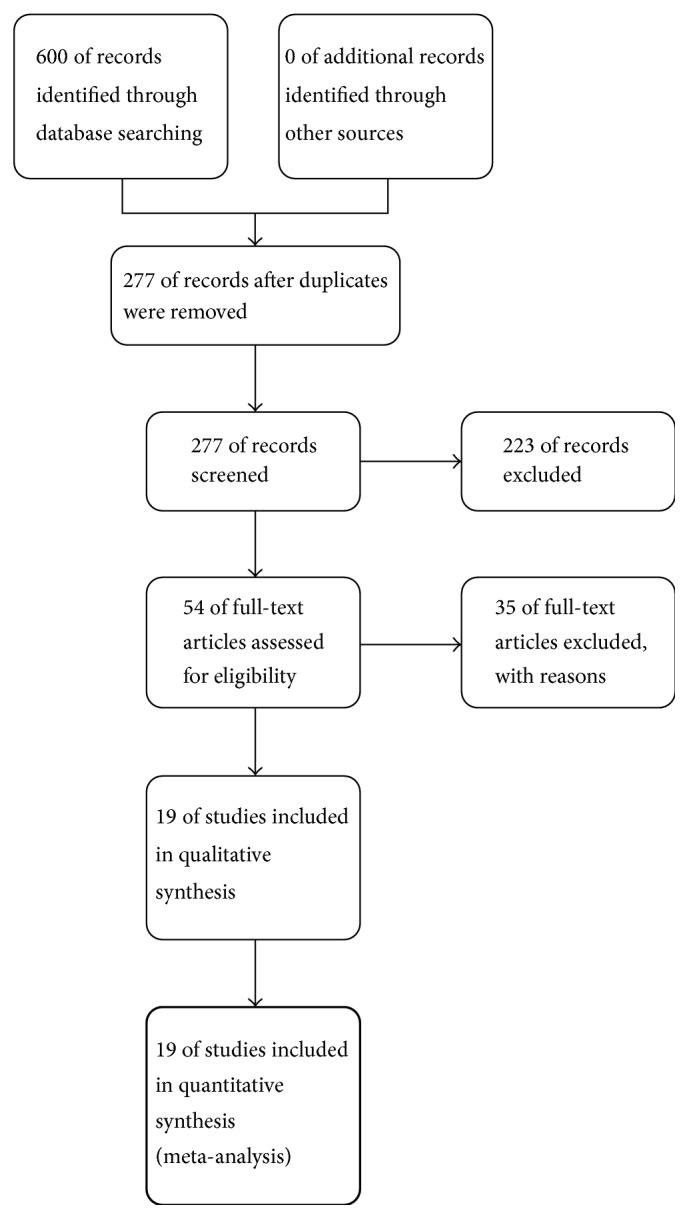
Study flow diagram.

We retrieved 600 records using the search strategy specified in our protocol. 223 records were discarded after reviewing the titles and/or abstracts. Thirty-five articles that initially appeared to meet the inclusion criteria were excluded with reasons: (i) not stable COPD (*n* = 24), (ii) not targeted comparators (*n* = 9), (iii) not targeted outcomes (*n* = 1), and (iv) full-text articles unavailable (*n* = 1). Thus, nineteen studies (1298 participants) finally met our criteria and were included in this review [7–25]. The study selection process was outlined in Figure 1.

### 3.2. Data Extraction and Assessment of Risk of Bias

**Figure 2:**
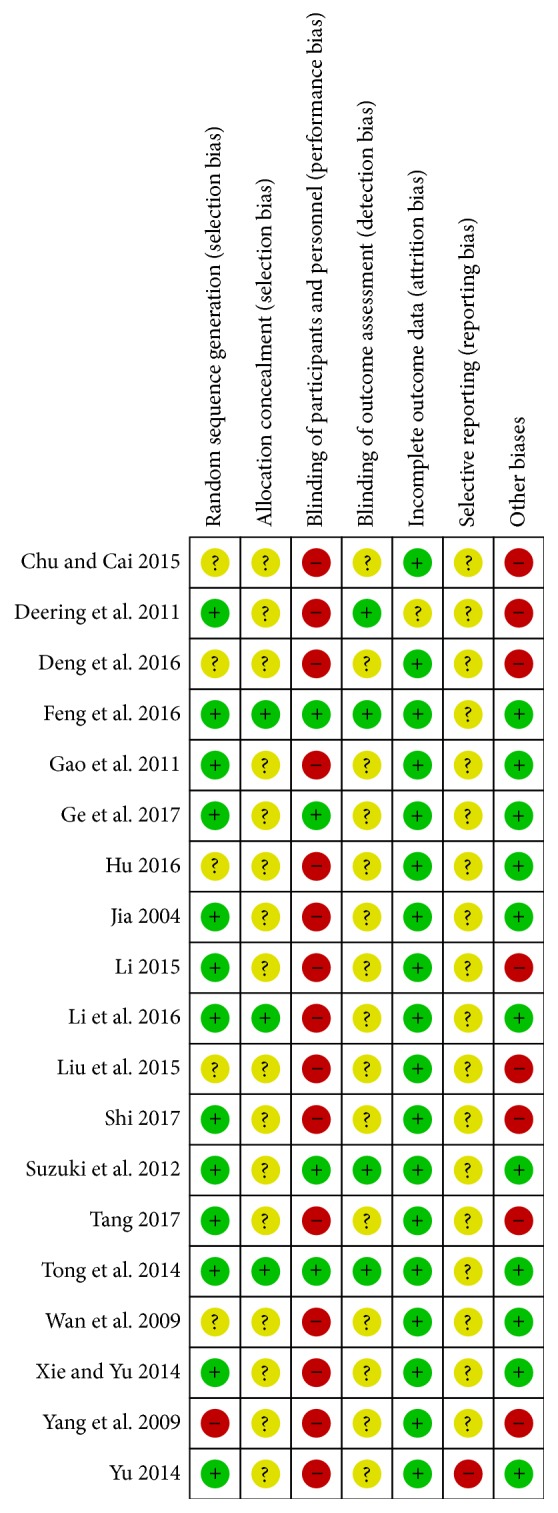
Risk of bias summary: review authors' judgements about each risk of bias item for each included study.

**Figure 3:**
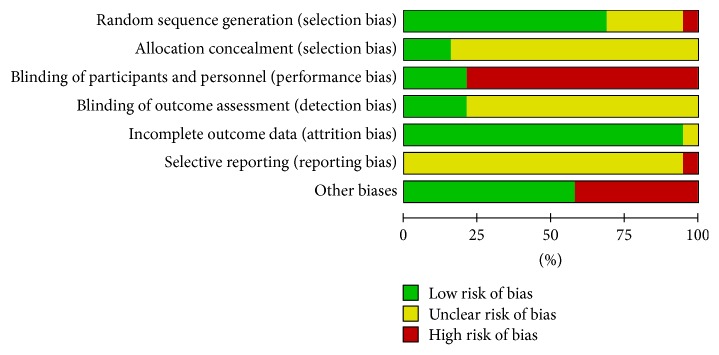
Risk of bias graph: review authors' judgements about each risk of bias item presented as percentages across all included studies.

A detailed description of the characteristics of included studies was outlined in Table 1. We determined the Cochrane “risk of bias” score for each study and this information was summarized in Table 2 and Figures 2 and 3.

### 3.3. Effects of Interventions

#### 3.3.1. 6MWD

**Figure 4:**
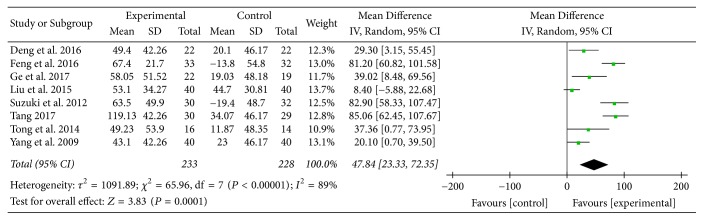
Experimental group versus control group, 6MWD.

Eight studies [9, 10, 16–18, 21, 23, 25] provided numerical data for 6MWD and were included in the meta-analysis. Analysis of the data indicated that there was heterogeneity (*χ* ^2^ = 65.96, *P* < 0.00001; *I* ^2^ = 89%); hence, a random-effect model was used. The pooled results showed that 6MWD in the experimental group improved more compared to the control group (MD: 47.84; 95% CI: 23.33 to 72.35; *Z* = 3.83, *P* = 0.0001) (Figure 4).

#### 3.3.2. SGRQ

**Figure 5:**
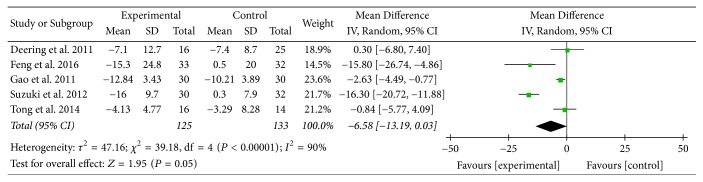
Experimental group versus control group, SGRQ total scores.

Five studies [8, 10, 11, 17, 18] provided numerical data for SGRQ total scores and were included in the meta-analysis. Analysis of the data indicated that there was heterogeneity (*χ* ^2^ = 39.18, *P* < 0.00001; *I* ^2^ = 90%); hence, a random-effect model was used. The pooled results showed that there was no significant improvement in SGRQ total scores between two groups (MD: −6.58; 95% CI: −13.19 to 0.03; *Z* = 1.95, *P* = 0.05) (Figure 5).

**Figure 6:**
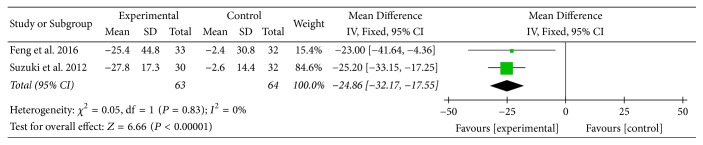
Experimental group versus control group, symptom domain scores of SGRQ.

Two studies [10, 17] provided numerical data for symptom domain scores of SGRQ and were included in the meta-analysis. Analysis of the data indicated that heterogeneity was not statistically significant (*χ* ^2^ = 0.05, *P* = 0.83; *I* ^2^ = 0%); hence, a fixed-effect model was used. The pooled results showed that symptom domain scores of SGRQ in the experimental group improved more compared to the control group (MD: −24.86; 95% CI: −32.17 to −17.55; *Z* = 6.66, *P* < 0.00001) (Figure 6).

**Figure 7:**
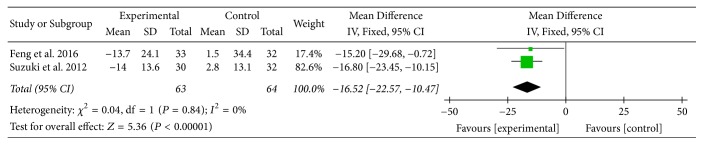
Experimental group versus control group, activity domain scores of SGRQ.

Two studies [10, 17] provided numerical data for activity domain scores of SGRQ and were included in the meta-analysis. Analysis of the data indicated that heterogeneity was not statistically significant (*χ* ^2^ = 0.04, *P* = 0.84; *I* ^2^ = 0%); hence, a fixed-effect model was used. The pooled results showed that activity domain scores of SGRQ in the experimental group improved more compared to the control group (MD: −16.52; 95% CI: −22.57 to −10.47; *Z* = 5.36, *P* < 0.00001) (Figure 7).

**Figure 8:**
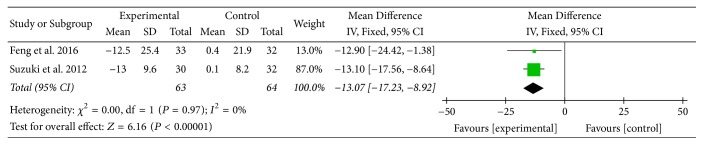
Experimental group versus control group, impact domain scores of SGRQ.

Two studies [10, 17] provided numerical data for impact domain scores of SGRQ and were included in the meta-analysis. Analysis of the data indicated that heterogeneity was not statistically significant (*χ* ^2^ = 0.00, *P* = 0.97; *I* ^2^ = 0%); hence, a fixed-effect model was used. The pooled results showed that impact domain scores of SGRQ in the experimental group improved more compared to the control group (MD: −13.07; 95% CI: −17.23 to −8.92; *Z* = 6.16, *P* < 0.00001) (Figure 8).

#### 3.3.3. FEV_1_

**Figure 9:**
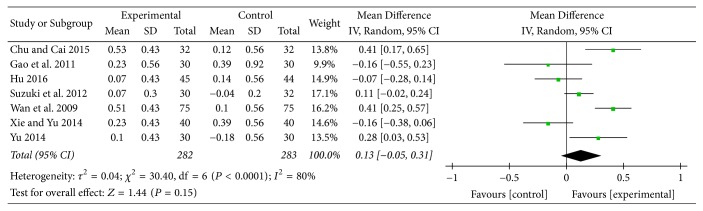
Experimental group versus control group, FEV_1_.

Seven studies [7, 11, 12, 17, 19, 20, 22] provided numerical data for FEV_1_ and were included in the meta-analysis. Analysis of the data indicated that there was heterogeneity (*χ* ^2^ = 30.40, *P* < 0.0001; *I* ^2^ = 80%); hence, a random-effect model was used. The pooled results showed that there was no significant improvement in FEV_1_ between two groups (MD: 0.13; 95% CI: −0.05 to 0.31; *Z* = 1.44, *P* = 0.15) (Figure 9).

#### 3.3.4. Effective Rate

**Figure 10:**
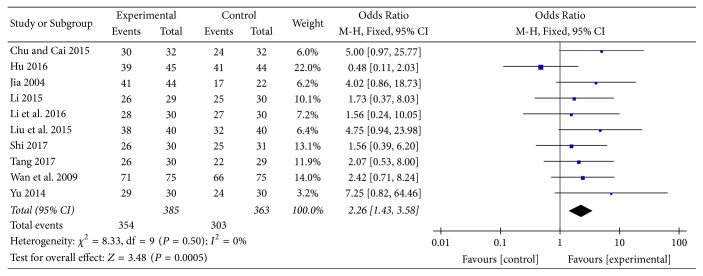
Experimental group versus control group, effective rate.

Ten studies [7, 12–16, 19, 22, 24, 25] provided categorical data for effective rate and were included in the meta-analysis. Analysis of the data indicated that heterogeneity was not statistically significant (*χ* ^2^ = 8.33, *P* = 0.50; *I* ^2^ = 0%); hence, a fixed-effect model was used. The pooled results showed that effective rate in the experimental group was higher compared to the control group (OR: 2.26; 95% CI: 1.43 to 3.58; *Z* = 3.48, *P* = 0.0005) (Figure 10).

#### 3.3.5. mMRC

Only one study [8] provided numerical data for mMRC scores; thus, the meta-analysis was not performed. Changes from baseline in mMRC scores in AT plus pulmonary rehabilitation (PR) group and PR group were −0.3 ± 0.5 and −0.3 ± 0.9, respectively. There was significant difference reported within AT plus PR group (*P* = 0.04).

### 3.4. Adverse Effects

Six studies [7, 14, 15, 17, 24, 25] provided information about adverse effects. Only one study [17] reported some minor adverse reactions during the trial including fatigue, subcutaneous hemorrhage, dizziness, and needle site pain, and the remaining 5 studies [7, 14, 15, 24, 25] reported no adverse effects.

### 3.5. Metaregression Analysis

We tried to perform a univariate metaregression analysis to explore potential associations between effect size and covariates of interest (publication year, region, intervention forms, sample size, and treatment period) (see Table 3). However, the results showed that there were no statistically significant associations among them, and this might be due to the insufficient number of studies included [32].

### 3.6. Subgroup Analysis (AT Adjunctive to Other Treatments versus Placebo or Sham Acupuncture Adjunctive to Other Treatments)

#### 3.6.1. 6MWD

**Figure 11:**
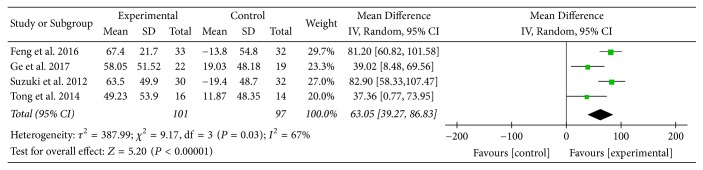
AT adjunctive to other treatments versus placebo or sham acupuncture adjunctive to other treatments, 6MWD.

Four studies [10, 17, 18, 23] provided numerical data for 6MWD and were included in the meta-analysis. Analysis of the data indicated that there was heterogeneity (*χ* ^2^ = 9.17, *P* = 0.03; *I* ^2^ = 67%); hence, a random-effect model was used. The pooled results showed that 6MWD in the experimental group improved more compared to the control group (MD: 63.05; 95% CI: 39.27 to 86.83; *Z* = 5.20, *P* < 0.00001) (Figure 11).

#### 3.6.2. SGRQ

**Figure 12:**
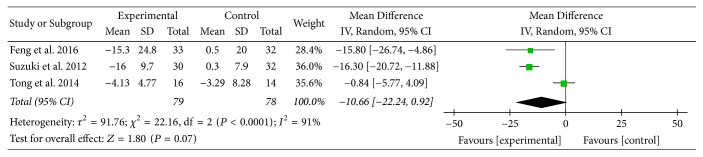
AT adjunctive to other treatments versus placebo or sham acupuncture adjunctive to other treatments, SGRQ total scores.

Three studies [10, 17, 18] provided numerical data for SGRQ total scores and were included in the meta-analysis. Analysis of the data indicated that there was heterogeneity (*χ* ^2^ = 22.16, *P* < 0.0001; *I* ^2^ = 91%); hence, a random-effect model was used. The pooled results showed that SGRQ total scores in the experimental group improved more compared to the control group (MD: −10.66; 95% CI: −22.24 to 0.92; *Z* = 1.80, *P* = 0.07) (Figure 12).

#### 3.6.3. FEV_1_

Only one study [17] provided numerical data for FEV_1_; thus, the meta-analysis was not performed. Changes from baseline in FEV_1_ in experimental group and control group were 0.07 ± 0.3 and −0.04 ± 0.2, respectively. However, the *P* values were not available.

### 3.7. Subgroup Analysis (AT Adjunctive to Other Treatments versus Other Treatments Alone)

#### 3.7.1. 6MWD

**Figure 13:**
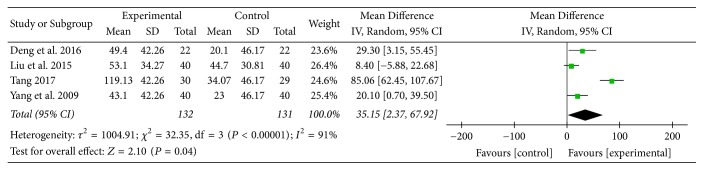
AT adjunctive to other treatments versus other treatments alone, 6MWD.

Four studies [9, 16, 21, 25] provided numerical data for 6MWD and were included in the meta-analysis. Analysis of the data indicated that there was heterogeneity (*χ* ^2^ = 32.35, *P* < 0.00001; *I* ^2^ = 91%); hence, a random-effect model was used. The pooled results showed that 6MWD in the experimental group improved more compared to the control group (MD: 35.15; 95% CI: 2.37 to 67.92; *Z* = 2.10, *P* = 0.04) (Figure 13).

#### 3.7.2. SGRQ

Only one study [8] provided numerical data for SGRQ total scores; thus, the meta-analysis was not performed. Compared to the control group (7.0 ± 14.9), both AT plus PR group and PR group demonstrated a significant change for SGRQ total scores (−7.1 ± 12.7, *P* = 0.01; −7.4 ± 8.7, *P* = 0.0006). However, there were no data available for symptom domain scores, activity domain scores, and impact domain scores of SGRQ.

#### 3.7.3. FEV_1_

**Figure 14:**
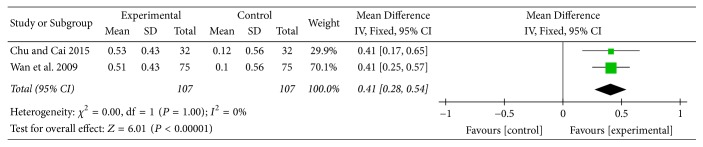
AT adjunctive to other treatments versus other treatments alone, FEV_1_.

Two studies [7, 19] provided numerical data for FEV_1_ and were included in the meta-analysis. Analysis of the data indicated that heterogeneity was not statistically significant (*χ* ^2^ = 0.00, *P* = 1.00; *I* ^2^ = 0%); hence, a fixed-effect model was used. The pooled results showed that FEV_1_ in the experimental group improved more compared to the control group (MD: 0.41; 95% CI: 0.28 to 0.54; *Z* = 6.01, *P* < 0.00001) (Figure 14).

#### 3.7.4. Effective Rate

**Figure 15:**
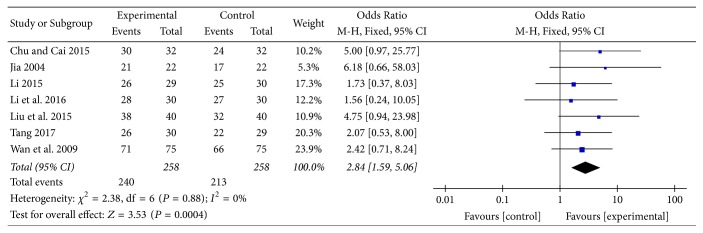
AT adjunctive to other treatments versus other treatments alone, effective rate.

Seven studies [7, 13–16, 19, 25] provided categorical data for effective rate and were included in the meta-analysis. Analysis of the data indicated that heterogeneity was not statistically significant (*χ* ^2^ = 2.38, *P* = 0.88; *I* ^2^ = 0%); hence, a fixed-effect model was used. The pooled results showed that effective rate in the experimental group was higher compared to the control group (OR: 2.84; 95% CI: 1.59 to 5.06; *Z* = 3.53, *P* = 0.0004) (Figure 15).

### 3.8. Reporting Biases

We did not investigate publication biases by funnel plot because each comparison included not more than 10 studies.

## 4. Discussion

This systematic review provided a detailed summary of the current evidences related to the efficacy and safety of AT for functional effects and quality of life in COPD patients.

6MWD is an important measure of functional exercise capacity of patients with COPD. The distance walked is associated with clinical outcomes such as hospitalization and mortality, and its changes are used to evaluate the efficacy of therapeutic interventions such as pulmonary rehabilitation, surgery, and pharmaceutical management [33, 34]. In this review, 6MWD in the experimental group improved more compared to the control group, and the MD was 47.84 meters, which was greater than 25 meters, the minimal clinically important difference (MCID) of 6MWD for COPD patients [34]. This result might indicate the potential of AT in improving exercise capacity of COPD patients. Two subgroup analyses supported this result as well, and the MD of 6MWD change was 63.05 meters and 35.15 meters, respectively.

SGRQ, another primary outcome measure in this review, is a well-established disease-specific instrument to measure quality of life for asthma and COPD. In this review, there was no statistically significant improvement in SGRQ total scores between two groups. However, MD of symptom domain scores, activity domain scores, and impact domain scores of SGRQ was 24.86 units, 16.52 units, and 13.07 units, respectively. Although there was no MCID available for each domain, each MD was at least three times greater than 4 units, the MCID for SGRQ total scores in COPD patients [35], and this might suggest the effect of AT on different aspects of health status in COPD patients. Subgroup analysis (AT adjunctive to other treatments versus placebo or sham acupuncture adjunctive to other treatments) supported these above results as well.

FEV_1_ is widely used by physicians in the diagnosis, classification, treatment, monitoring, and establishing prognosis for COPD patients. In this review, there was no statistically significant improvement in FEV_1_ between two groups. However, subgroup analysis (AT adjunctive to other treatments versus other treatments alone) showed MD of FEV_1_ change was 410 mL, which was four times greater than 100 mL, the MCID of FEV_1_ for COPD patients [36]. And this result might suggest the potential of AT in improving pulmonary function in COPD patients.

mMRC is a major instrument to measure breathlessness. In this review, since mMRC scores were only available in one study, the meta-analysis was not performed. According to this study, change from baseline in mMRC scores in AT plus PR group and PR group was 0.3 units in both, and it was reported that there was significant difference within AT plus PR group. However, it was limited to support the effect of AT in improving breathlessness in COPD patients.

Effective rate, an important outcome measure in clinical studies of Chinese medicine, was also evaluated. In this review, effective rate in the experimental group was higher compared to the control group; to some extent, this might suggest that AT was a more effective treatment compared to other treatments. Importantly, subgroup analysis (AT adjunctive to other treatments versus other treatments alone) also supported this result with OR of 2.84.

Adverse effects were poorly reported in included studies. One study reported some minor adverse effects, and 5 studies reported no adverse effects. This might indicate the safety of AT for COPD patients.

There were some limitations in this study. Firstly, methodological quality of the included studies was generally low. For example, most of the included studies had high risk of performance bias. Secondly, most analysis of the data in the meta-analysis indicated that there was heterogeneity. Thirdly, there were various intervention forms of AT, which might make it difficult to evaluate the efficacy of AT alone. Finally, some resources with language other than English and Chinese might not be included in this review.

## 5. Conclusions

AT may be effective and safe in improving functional effects and quality of life in COPD patients. Besides, AT may also improve pulmonary function of COPD patients. Evidences are inadequate to support the potential of AT in improving breathlessness of COPD patients. These evidences may be useful to clinicians, patients, and health policy-makers with regard to application of AT in COPD. However, further high-quality RCTs are needed to confirm the efficacy and safety of AT for COPD patients.

## Figures and Tables

**Table 1:** Characteristics of included studies.

Study	Country	Study design	Participants	Interventions	Outcomes	Notes
Chu and Cai [7] 2015	China	RCT, 2 arms	*Participant status:* Gender (M/F): EG: 15/17, CG: 14/18 Age (range, years): from 46 to 80 FEV_1_% predicted: EG: 56.83 ± 8.96; CG: 52.53 ± 13.96 Course of disease (range): from 5 months to 20 years *Participants randomly assigned:* 64 participants were randomly assigned Analyzed: EG: 32 CG: 32	EG: AT + Chinese medicine + western medicine CG: Chinese medicine + western medicine Duration: treatment for 90 days; follow-up for 6 months	Spirometry, acute exacerbation frequency, effective rate, adverse effects	

Deering et al. [8] 2011	Ireland	RCT, 3 arms	*Participant status:* Gender (M/F): PR group: 11/14; AT + PR group: 8/8; CG: 12/7 Age (years): PR group: 67.7 ± 5.3; AT + PR group: 65.1 ± 9.7; CG: 68.6 ± 5.5 FEV_1_% predicted: PR group: 48.5 ± 16.1; AT + PR group: 48.8 ± 22.7; CG: 45.8 ± 18.3 *Participants randomly assigned:* 60 participants were randomly assigned Analyzed: AT + PR group: 16 PR group: 25	AT + PR group: AT + PR PR group: PR alone CG: no intervention Duration: treatment for 7 weeks; follow-up for 3 months	Body mass index, mMRC, the modified Borg dyspnea score, systemic inflammation, spirometry, total energy expenditure, physical activity duration, metabolic equivalents, steps per day, sleep time/efficiency, incremental shuttle walk test, SGRQ, EQ-5D	Control group was not used in the analysis

Deng et al. [9] 2016	China	RCT, 2 arms	*Participant status:* Gender (M/F): EG: 13/9, CG: 15/7 Age (years): EG: 57.1 ± 5.9; CG: 58.5 ± 6.5 FEV_1_% predicted: EG: 52.4 ± 2.9; CG: 51.5 ± 2.7 Course of disease (years): EG: 6.4 ± 2.5; CG: 6.3 ± 2.1 *Participants randomly assigned:* 44 participants were randomly assigned Analyzed: EG: 22 CG: 22	EG: abdominal AT + conventional therapy CG: conventional therapy alone Duration: treatment for 24 weeks	Annual hospital stay, annual acute exacerbation frequency, oxygen saturation level, spirometry, 6MWD	

Feng et al. [10] 2016	China	RCT, 2 arms	*Participant status:* Gender (M/F): EG: 33/3, CG: 31/5 Age (years): EG: 67.8 ± 5.4; CG: 67.1 ± 6.1 FEV_1_% predicted: EG: 47.3 ± 17.1; CG: 43.9 ± 16.8 *Participants randomly assigned:* 72 participants were randomly assigned Analyzed: EG: 33 CG: 32	EG: AT + daily medication CG: Sham acupuncture + daily medication Duration: treatment 3 times weekly for 8 weeks	6MWD, modified Borg scale score before and after 6MWT, oxygen saturation during the 6MWT, spirometry, SGRQ	

Gao et al. [11] 2011	China	RCT, 2 arms	*Participant status:* Gender (M/F): EG: 13/17, CG: 12/18 Age (years): EG: 64.87 ± 8.73; CG: 65.25 ± 10.66 FEV_1_% predicted: EG: 45.88 ± 5.05; CG: 43.54 ± 6.29 Course of disease (years): EG: 10.31 ± 5.82; CG: 10.78 ± 5.53 *Participants randomly assigned:* 61 participants were randomly assigned Analyzed: EG: 30 CG: 30	EG: warm AT CG: drug therapy Duration: treatment for 8 weeks	Spirometry, clinical symptoms, SGRQ	

Hu [12] 2016	China	RCT, 2 arms	*Participant status:* Gender (M/F): not described Age (years): not described FEV_1_% predicted: not described Course of disease (years): not described *Participants randomly assigned:* 89 participants were randomly assigned Analyzed: EG: 45 CG: 44	EG: warm AT + conventional therapy CG: spreading moxibustion + conventional therapy Duration: treatment for 2 months	Effective rate, spirometry	

Jia [13] 2004	China	RCT, 3 arms	*Participant status:* Gender (M/F): AT group: 9/13; PR group: 8/14; AT + PR group: 10/12 Age (years): AT group: 61.0 ± 32.6; PR group: 60.0 ± 34.8; AT + PR group: 61.0 ± 33.2 FEV_1_% predicted: AT group: 70.9 ± 0.2; PR group: 71.1 ± 0.1; AT + PR group: 70.6 ± 0.7 Course of disease (years): AT group: 11.7 ± 8.9; PR group: 12.1 ± 7.2; AT + PR group: 11.6 ± 9.0 *Participants randomly assigned:* 66 participants were randomly assigned Analyzed: AT group: 22 PR group: 22 AT + PR group: 22	AT group: AT + conventional drugs PR group: PR + conventional drugs AT + PR group: AT + PR + conventional drugs Duration: treatment for 100 days	Effective rate, spirometry	Data from AT group and AT + PR group were combined in the analysis

Li [14] 2015	China	RCT, 2 arms	*Participant status:* Gender (M/F): EG: 19/10, CG: 17/13 Age (years): EG: 57.21 ± 6.68; CG: 55.80 ± 7.23 FEV_1_% predicted: EG: 66.28 ± 6.86; CG: 65.16 ± 6.16 Course of disease (years): EG: 10.38 ± 4.90; CG: 10.70 ± 4.88 *Participants randomly assigned:* 60 participants were randomly assigned Analyzed: EG: 29 CG: 30	EG: warm AT + drug therapy CG: drug therapy alone Duration: treatment for 1 month	Safety indicators, CAT, spirometry, clinical symptoms, effective rate, adverse effects	

Li et al. [15] 2016	China	RCT, 2 arms	*Participant status:* Gender (M/F): two groups were reported to be comparable in gender. Age (years): two groups were reported to be comparable in age. FEV_1_% predicted: EG: 53.1 ± 10.9; CG: 51.9 ± 11.4 *Participants randomly assigned:* 60 participants were randomly assigned Analyzed: EG: 30 CG: 30	EG: AT + Chinese medicine + western medicine CG: Chinese medicine + western medicine Duration: treatment for 3 weeks	Clinical symptoms, CAT, spirometry, effective rate, adverse effects	

Liu et al. [16] 2015	China	RCT, 2 arms	*Participant status:* Gender (M/F): EG: 24/16, CG: 26/14 Age (years): EG: 58.3 ± 12.4; CG: 63.2 ± 10.7 FEV_1_% predicted: EG: 35.71 ± 7.28; CG: 36.42 ± 6.42 *Participants randomly assigned:* 80 participants were randomly assigned Analyzed: EG: 40 CG: 40	EG: AT + drug therapy CG: drug therapy alone Duration: treatment for 3 months	Clinical signs and symptoms, 6MWD, spirometry, effective rate	

Suzuki et al. [17] 2012	Japan	RCT, 2 arms	*Participant status:* Gender (M/F): EG: 31/3, CG: 32/2 Age (years): EG: 72.7 ± 6.8; CG: 72.5 ± 7.4 FEV_1_% predicted: EG: 44.5 ± 16.3; CG: 48.0 ± 16.5 *Participants randomly assigned:* 68 participants were randomly assigned Analyzed: EG: 30 CG: 32	EG: AT + daily medication CG: placebo acupuncture + daily medication Duration: treatment once a week for 12 weeks	6MWD, modified Borg scale score before and after 6MWT, oxygen saturation during the 6MWT, spirometry, SGRQ, arterial blood gas, maximum inspiratory mouth pressure, maximum expiratory mouth pressure, range of motion in the rib cage, body mass index, serum prealbumin levels, MRC score, adverse reactions	

Tong et al. [18] 2014	China	RCT, 2 arms	*Participant status:* Gender (M/F): EG: 15/1, CG: 12/2 Age (years): EG: 64 ± 6; CG: 67 ± 6 FEV_1_% predicted: EG: 41.72 ± 17.95; CG: 36.16 ± 16.29 *Participants randomly assigned:* 30 participants were randomly assigned Analyzed: EG: 16 CG: 14	EG: AT + aerobic exercise CG: placebo acupuncture + aerobic exercise Duration: treatment for 5 weeks	6MWD, spirometry, maximum oxygen uptake, exercise time, SGRQ	

Wan et al. [19] 2009	China	RCT, 2 arms	*Participant status:* Gender (M/F): EG: 47/28, CG: 45/30 Age (years): EG: 62.40 ± 8.56; CG: 61.80 ± 10.10 FEV_1_% predicted: EG: 48.07 ± 12.77; CG: 47.27 ± 14.02 Course of disease (years): EG: 7.73 ± 3.80; CG: 7.70 ± 2.92 *Participants randomly assigned:* 150 participants were randomly assigned Analyzed: EG: 75 CG: 75	EG: AT + Chinese medicine CG: Chinese medicine alone Duration: treatment for 36 days	Effective rate, spirometry	

Xie and Yu [20] 2014	China	RCT, 2 arms	*Participant status:* Gender (M/F): EG: 22/18, CG: 18/22 Age (years): EG: 68.9 ± 8.7; CG: 68.5 ± 9.6 FEV_1_% predicted: EG: 45.89 ± 5.06; CG: 43.55 ± 6.30 Course of disease (years): EG: 11.8 ± 6.5; CG: 12.3 ± 5.5 *Participants randomly assigned:* 80 participants were randomly assigned Analyzed: EG: 40 CG: 40	EG: warm AT CG: drug therapy Duration: treatment for 8 weeks	Spirometry, symptom scores	

Yang et al. [21] 2009	China	RCT, 2 arms	*Participant status:* Gender (M/F): EG: 26/14, CG: 28/12 Age (range, years): EG: from 49 to 78; CG: from 45 to 77 FEV_1_% predicted: EG: 53.5 ± 19.2; CG: 54.3 ± 22.6 Course of disease (mean, years): EG: 6.9; CG: 7.1 *Participants randomly assigned:* 80 participants were randomly assigned Analyzed: EG: 40 CG: 40	EG: AT + PR CG: PR alone Duration: treatment for 40 days	COPD quality of life questionnaire, 6MWD, spirometry	

Yu [22] 2014	China	RCT, 2 arms	*Participant status:* Gender (M/F): EG: 18/12, CG: 17/13 Age (years): EG: 63.0 ± 8.5; CG: 62.0 ± 7.6 FEV_1_% predicted: EG: 50.23 ± 2.56; CG: 51.33 ± 2.43 Course of disease (years): EG: 8.9 ± 3.7; CG: 8.4 ± 3.5 *Participants randomly assigned:* 60 participants were randomly assigned Analyzed: EG: 30 CG: 30	EG: warm AT + function training CG: conventional drug therapy + function training Duration: treatment for 3 months	Arterial blood gas, spirometry, SGRQ, effective rate	

Ge et al. [23] 2017	China	RCT, 2 arms	*Participant status:* Gender (M/F): EG: 23/1, CG: 15/5 Age (years): EG: 65 ± 6; CG: 65 ± 7 FEV_1_% predicted: EG: 40.76 ± 16.36; CG: 40.53 ± 17.40 Course of disease (years): EG: 9.1 ± 5.5; CG: 8.6 ± 6.8 *Participants randomly assigned:* 44 participants were randomly assigned Analyzed: EG: 22 CG: 19	EG: AT + conventional drugs + aerobic exercise CG: placebo acupuncture + conventional drugs + aerobic exercise Duration: treatment for 14 times	Body mass index, average distance and average maximum heart rate during bicycle exercise, 6MWD, maximum power and maximum heart rate during exercise cardiopulmonary function test, spirometry	

Shi [24] 2017	China	RCT, 2 arms	*Participant status:* Gender (M/F): EG: 18/12, CG: 17/14 Age (years): EG: 57.77 ± 6.54; CG: 55.90 ± 6.86 FEV_1_% predicted: EG: 64.11 ± 5.79; CG: 65.85 ± 6.86 Course of disease (years): EG: 11.43 ± 4.37; CG: 11.68 ± 3.64 *Participants randomly assigned:* 66 participants were randomly assigned Analyzed: EG: 30 CG: 31	EG: AT CG: western medicine Duration: treatment for 2 months	CAT, spirometry, clinical symptoms, effective rate, safety indicators	

Tang [25] 2017	China	RCT, 2 arms	*Participant status:* Gender (M/F): EG: 19/11, CG: 18/13 Age (years): EG: 57.47 ± 5.33; CG: 56.59 ± 6.34 Course of disease (years): EG: 10.07 ± 2.20; CG: 10.55 ± 2.10 *Participants randomly assigned:* 64 participants were randomly assigned Analyzed: EG: 30 CG: 29	EG: AT + western medicine CG: western medicine alone Duration: treatment for 2 months	CAT, clinical symptoms, 6MWT, effective rate, safety indicators	

RCT: randomized controlled trial, EG: experimental group, CG: control group, AT: acupuncture therapy, PR: pulmonary rehabilitation, FEV_1_: forced expiratory volume in 1 second, 6MWT/MWD: six-minute walk test/distance, SGRQ: St. George's Respiratory Questionnaire, mMRC: modified Medical Research Council dyspnea scale, MRC: Medical Research Council dyspnea scale, CAT: COPD assessment test.

**Table 2:** Risks of bias of included studies.

Study	Random sequence generation	Allocation concealment	Blinding of participants and personnel	Blinding of outcome assessment	Incomplete outcome data	Selective reporting	Other biases
Chu and Cai [7] 2015	U	U	H	U	L	U	H
Deering et al. [8] 2011	L	U	H	L	U	U	H
Deng et al. [9] 2016	U	U	H	U	L	U	H
Feng et al. [10] 2016	L	L	L	L	L	U	L
Gao et al. [11] 2011	L	U	H	U	L	U	L
Hu [12] 2016	U	U	H	U	L	U	L
Jia [13] 2004	L	U	H	U	L	U	L
Li [14] 2015	L	U	H	U	L	U	H
Li et al. [15] 2016	L	L	H	U	L	U	L
Liu et al. [16] 2015	U	U	H	U	L	U	H
Suzuki et al. [17] 2012	L	U	L	L	L	U	L
Tong et al. [18] 2014	L	L	L	L	L	U	L
Wan et al. [19] 2009	U	U	H	U	L	U	L
Xie and Yu [20] 2014	L	U	H	U	L	U	L
Yang et al. [21] 2009	H	U	H	U	L	U	H
Yu [22] 2014	L	U	H	U	L	H	L
Ge et al. [23] 2017	L	U	L	U	L	U	L
Shi [24] 2017	L	U	H	U	L	U	H
Tang [25] 2017	L	U	H	U	L	U	H

*Notes*. Quality assessment based on the Cochrane tools for assessing risk of bias. L: low (low risk of bias), H: high (high risk of bias), U: unclear (uncertain risk of bias).

**Table 3:** Univariate metaregression analysis of covariates of interest.

Covariates of interest	Coefficient	Standard error (SE)	*t*	*P*	95% confidence intervals (CI)	
					Lower limit	Upper limit
Six-minute walk distance						
Publication year	2.812	4.479	0.63	0.553	−8.148	13.773
Region	*∗*	*∗*	*∗*	*∗*	*∗*	*∗*
Intervention forms	26.947	21.520	1.25	0.257	−25.710	79.603
Sample size	−0.236	0.716	−0.33	0.753	−1.988	1.516
Treatment period	−0.115	0.292	−0.39	0.707	−0.829	0.599
*SGRQ*						
Publication year	−1.634	2.107	−0.78	0.495	−8.340	5.072
Region	8.595	9.520	0.90	0.433	−21.702	38.891
Intervention forms	4.605	4.389	1.05	0.371	−9.363	18.573
Sample size	−0.382	0.225	−1.70	0.188	−1.097	0.334
Treatment period	−0.342	0.139	−2.47	0.090	−0.783	0.099
*FEV*_*1*_						
Publication year	−0.028	0.043	−0.65	0.544	−0.139	0.083
Region	*∗*	*∗*	*∗*	*∗*	*∗*	*∗*
Intervention forms	−0.086	0.088	−0.98	0.371	−0.313	0.140
Sample size	0.003	0.003	0.83	0.447	−0.005	0.011
Treatment period	0.002	0.005	0.48	0.654	−0.011	0.016
*Effective rate*						
Publication year	−0.064	0.060	−1.07	0.315	−0.203	0.074
Region	*∗*	*∗*	*∗*	*∗*	*∗*	*∗*
Intervention forms	−0.117	0.301	−0.39	0.707	−0.812	0.577
Sample size	−0.002	0.008	−0.22	0.830	−0.021	0.017
Treatment period	0.015	0.010	1.54	0.163	−0.008	0.037

*Note*. 6MWD: six-minute walk test/distance, SGRQ: St. George's Respiratory Questionnaire. ^*∗*^The region was the same.

## Additional Points

*Registration Number*. This article is registered with PROSPERO 2016 CRD42016054335 (available from http://www.crd.york.ac.uk/PROSPERO/display_record.php?ID=CRD42016054335).

## Supplementary Materials

Supplementary Materials The methods for this study had been developed according to the recommendations of the Preferred Reporting Items for Systematic Reviews and Meta-Analyses (PRISMA) statement.
